# Expression of plasma IFN signaling-related miRNAs during acute SARS-CoV-2 infection and its association with RBD-IgG antibody response

**DOI:** 10.1186/s12985-021-01717-7

**Published:** 2021-12-07

**Authors:** Jing Wu, Xingxiang Liu, Jianguo Shao, Yuanyuan Zhang, Renfei Lu, Hong Xue, Yunfang Xu, Lijuan Wang, Hui Zhou, Lugang Yu, Ming Yue, Chen Dong

**Affiliations:** 1 Department of Epidemiology and Statistics, School of Public Health, Medical College of Soochow University, Soochow, China; 2 Department of Clinical Laboratory, Huai’an Fourth People’s Hospital, Huai’an, China; 3 Nantong Third People’s Hospital, Nantong, China; 4 Suzhou Industrial Park Centers for Disease Control and Prevention, Soochow, China; 5 Department of Infectious Diseases, The First Affiliated Hospital of Nanjing Medical University, Nanjing, China

**Keywords:** SARS-CoV-2, COVID-19, MicroRNA, RBD-IgG, IFN-I signaling pathway

## Abstract

**Background:**

Coronavirus disease 2019 (COVID-19) is a huge challenge worldwide. Although previous studies have suggested that type I interferon (IFN-I) could inhibit the virus replication, the expression characteristics of IFN-I signaling-related miRNAs (ISR-miRNAs) during acute severe acute respiratory syndrome coronavirus-2 (SARS-CoV-2) infection and its relationship with receptor-binding domain (RBD) IgG antibody response at the recovery phase remain unclear.

**Methods:**

Expression profiles of 12 plasma ISR-miRNAs in COVID-19 patients and healthy controls were analyzed using RT-qPCR. The level of RBD-IgG antibody was determined using the competitive ELISA. Spearman correlation was done to measure the associations of plasma ISR-miRNAs with clinical characteristics during acute SARS-CoV-2 infection and RBD-IgG antibody response at the recovery phase.

**Results:**

Compared with the healthy controls, COVID-19 patients exhibited higher levels of miR-29b-3p (Z = 3.15, *P* = 0.002) and miR-1246 (Z = 4.98, *P* < 0.001). However, the expression of miR-186-5p and miR-15a-5p were significantly decreased. As the results shown, miR-30b-5p was negatively correlated with CD4 + T cell counts (r = − 0.41, *P* = 0.027) and marginally positively correlated with fasting plasma glucose in COVID-19 patients (r = 0.37, *P* = 0.052). The competitive ELISA analysis showed the plasma level of miR-497-5p at the acute phase was positively correlated with RBD-IgG antibody response (r = 0.48, *P* = 0.038).

**Conclusions:**

Our present results suggested that the expression level of ISR-miRNAs was not only associated with acute SARS-CoV-2 infection but also with RBD-IgG antibody response at the recovery phase of COVID-19. Future studies should be performed to explore the biological significance of ISR-miRNAs in SARS-CoV-2 infection.

**Supplementary Information:**

The online version contains supplementary material available at 10.1186/s12985-021-01717-7.

## Background

Coronavirus disease 2019 (COVID-19), caused by severe acute respiratory syndrome coronavirus-2 (SARS-CoV-2), has brought a huge challenge to more than 200 countries and regions [1]. According to the COVID-19 Data Repository by The Center for Systems Science and Engineering at Johns Hopkins University, as of 6 August 2021, there have been more than 200 million laboratory-confirmed cases of COVID-19 with 4,289,796 deaths [2].

Type I interferon (IFN-I) exists in vertebrates and triggers the Januskinase/signal transducer and activator of transcription (JAK/STAT) signaling pathway with subsequent induction of IFN-stimulated genes (ISGs) [3]. Previously, mounting studies have shown that IFN-I could affect the life cycle of the virus by regulating the expression of related microRNAs (miRNAs). For example, Aboulnasr et al*.* reported that IFN-α/β could induce the expression of miR-122 in hepatocytes. However, the reduction of miR-122 expression level could weaken the effect of IFN-a/β in inhibiting hepatitis C virus (HCV) replication [4]. On the other hand, many viruses develop strategies to alter miRNA expression, thereby inhibiting the activity of IFN-I signaling pathway. For example, the results from the sequence alignment indicated that the presence of putative miRNA target sites for the IFN-I-induced miRNAs located in strictly conserved areas of the HCV genome. Pedersen et al*.* further confirmed that the expression levels of several miRNAs including miR-196, miR-296, miR-351, miR-431 and miR-448 might be affected by binding to the complementary sequences in HCV genome [5].

During the past year, several thousand studies have investigated the epidemiologic, clinical, biological and radiological characteristics of SARS-CoV-2 infection [6–8]. However, the effects of IFN-I signaling-related miRNAs (ISR-miRNAs) on the virus infection have not yet been fully understood. Based on the prediction results from miRDB and miRPathDB, twelve candidate binding sites for ISR-miRNAs in the genome of SARS-CoV-2 have been identified [9, 10]. In this study, the expression characteristics of these ISR-miRNAs during acute SARS-CoV-2 infection and their associations with receptor-binding domain (RBD) IgG antibody response at the recovery phase were further analyzed.

## Materials and methods

### Study participants and data collection

Between January 2020 and May 2020, 29 COVID-19 patients at the acute phase of COVID-19 and 29 gender and age (± 5 years) matched healthy controls were recruited from Huai’an Fourth hospital. All COVID-19 patients (26 mild and three severe cases) were positive for SARS-CoV-2 RNA in pharyngeal swab specimens and diagnosed according to the “New Coronavirus Pneumonia Prevention and Control Program (5^th^ version)” published by the National Health Commission of China [11]. In addition, the healthy controls were laboratory-confirmed cases on the basis of negative qRT-PCR results for SARS-CoV-2 in swab samples. In this study, patients who co-infected with other viruses were excluded. This study was approved by the ethics committee of Huai’an Fourth Hospital, Huai’an, China (HASY2020004), and conducted in accordance with the Declaration of Helsinki. All participants signed informed consent forms.

The demographic characteristics of COVID-19 patients and controls, including age, gender and exposure history were collected by face-to-face interview. In addition, the data about clinical signs, symptoms, potential comorbidities and laboratory indices during the acute phase of infection were extracted retrospectively from the electronic medical record system. The incubation period of the disease was defined as the time from exposure to the onset of illness, which was estimated among the patients who could provide the exact date of close contact with individuals confirmed or suspected SARS-CoV-2 infection.

### Blood sample collection

Five milliliters of blood samples were collected from each COVID-19 patient within 24 h of hospitalization (acute phase) and three months after discharge (recovery phase), respectively. In addition, five milliliters of fasting blood samples were also collected from the recruited age- and gender-matched healthy controls. All blood samples were centrifuged at 3000 g for 10 min. Plasmas were separated and inactivated in a water bath at 56 °C for 30 min, and then stored at -80 °C as quickly as possible.

### ISR-miRNA selection

The complete genome of SARS-CoV-2 strain (NC_045512.2) retrieved from the GenBank database was used as a reference sequence. The miRDB (http://www.mirdb.org/) was firstly used to identify miRNAs which can target the virus genome. The miRNAs with more than 95 of the target score were primarily included [12, 13]. Then, miRPathDB (https://mpd.bioinf.uni-sb.de/overview.html) was used to identify miRNAs related to the JAK-STAT pathway [14]. After conducted a systematic literature review to identify the ISR-miRNAs using the following terms “JAK”, “STAT”, and “JAK/STAT” in PubMed, twelve miRNAs were finally selected for the present analysis: let-7c-5p, miR-15a-5p, miR-15b-5p, miR-29b-3p, miR-30b-5p, miR-146b-3p, miR-148a-3p, miR-186-5p, miR-409-3p, miR-497-5p, miR-548c-5p and miR-1246. The detailed information about the selected ISR-miRNAs was listed in Additional file 1: Table S1.

### Plasma RNA extraction

Total RNA was isolated from each plasma sample with a commercial RNA extraction and purification kit (MACHEREYNAGEL SA, France) according to the manufacturer’s protocol. To warrant consistency in the experimental procedures, exogenous cel-miR-39 (mi*DETECT*™ miRNA External Control, RiboBio, China) was spiked into each sample before RNA extraction and used as an internal control for normalizing miRNA expression levels. The concentration of RNA was measured at OD260/280 by a NanoDrop ND-1000 spectrophotometer (Thermo Scientific, Wilmington, Delaware) and kept at − 20 °C until use.

### ISR-miRNA quantitation analysis

ISR-miRNA quantitation analysis was performed on a Q6 Real-Time System (Applied Biosystems) using the SYBR Green-based real-time detection method. The mi*DETECT*A Track™ miRNA qRT-PCR Starter Kit, the upstream and downstream primers of selected ISR-miRNAs were ordered from RiboBio Corporation (Guangzhou, China) [15]. The product numbers of the primers for ISR-miRNAs quantitation were showed in Additional file 2: Table S2. The reaction system for ISR-miRNA Poly(A) tailing contained 1 μg of total small RNA, 2 μL 5 × Poly(A) polymerase buffer, 1 μL Poly(A) polymerase and RNase-free water up to 10 μL. The reaction system for reverse transcription contained 4 μL RTase mix, 4 μL 5 × RTase buffer, 2 μL Uni-RT Primer and 10 μL Poly(A) Tailing product. 20 μL reaction system for real-time quantitative PCR containing 0.5 μL Forward Primer (10 μM), 0.5 μL Uni-Reverse Primer (10 μM), 10 μL 2 × SYBR Green Mix, 0.04 μL ROX Reference Dye, 2 μL cDNA and RNase-free water. The cycle threshold (CT value) was defined as the number of cycles required for the fluorescent signal to cross the threshold. As described above, cel-miR-39 was selected as an endogenous control for miRNA expression analysis. The expression of ISR-miRNA relative to cel-miR-39 miRNA was reported as dCT (ΔCT), which was calculated by subtracting the Ct of cel-miR-39 from the Ct of target ISR-miRNA. The relative quantitative of each ISR-miRNA between the two groups was calculated using 2^− (ΔCT)^.

### Competitive ELISA for RBD-IgG antibody detection

The competitive ELISA steps were carried out using “RBD-IgG antibody detection kit” (Beijing, China) according to the manufacturer’s introduction. Briefly, 96-well Corning Costar high binding plates were coated with SARS-CoV-2 spike RBD protein at a concentration of 0.1 μg per well. For the RBD-IgG antibody measurement, 50 μL of 1:50 diluted plasma sample and 50 μL of HRP-conjugated ACE2-mFc (0.2 μg/mL) were added into each well. Meanwhile, two negative, two positive plasma controls and two blank wells were included on each plate, respectively. After incubated for 30 min, the plate was washed three times and TMB substrate solution was added. The reaction was stopped after 15 min by the addition of 2 M H_2_SO_4_. The OD (optical density) at 450 nm was measured with an EMax Plus microplate reader (Molecular Devices, San Jose, CA). Results were expressed as percent inhibition (PI), calculated using the following formula: PI = 100% × [1 – (sample OD-blank OD)/(negative control OD-blank OD)]. The sample with more than 25% of PI value was considered as anti-RBD IgG positive.

### Statistical analysis

All statistical analyses were performed using SAS software (version 9.4). Two-sided *P* < 0.05 was considered statistically significant. Differences in the relative expression levels of ISR-miRNAs between COVID-19 patients and healthy controls were compared via Mann–Whitney U test. Spearman correlation was done to measure the correlations between the plasma ISR-miRNA level and clinically relevant parameters at the acute phase of COVID-19, as well as the RBD-IgG antibody levels at the recovery phase.

## Results

### Demographic and clinical characteristics of participants

As the results shown in Table 1, the mean age of COVID-19 patients and healthy controls was 47.45 ± 15.72 and 48.34 ± 13.50 years, respectively. Compared with healthy controls, patients with COVID-19 presented lower lymphocyte counts (Z = 3.86, *P* < 0.001) and platelet counts (Z = 2.80, *P* = 0.005). In addition, three, nine and one COVID-19 patients had preexisting diabetes, hypertension and renal insufficiency, respectively. The most common symptom at the onset of illness was fever (75.86%). The median incubation period of the disease was 5 days and the virus nucleic acid test turned negative about 7 days after admission.

**Table 1:** Demographic and clinical characteristics of included participants

Characteristics	Healthy controls (n = 29)	COVID-19 (n = 29)	*t*/Z/χ^2^	*P*
Age, years	48.34 ± 13.50	47.45 ± 15.72	0.23	0.817
Male, n (%)	14 (48.28)	17 (58.62)	0.62	0.430
BMI, kg/cm^2^	24.41 ± 4.33	26.40 ± 3.84	1.85	0.070
Signs and symptoms				
Fever	NA	22 (75.86)		
Cough	NA	14 (48.27)		
Myalgia or fatigue	NA	2 (6.90)		
Expectoration	NA	2 (6.90)		
Headache	NA	1 (3.44)		
Oppression in chest	NA	2 (6.90)		
Incubation period (days)	NA	5.0 (4.0, 7.0)		
Days from first admission to transfer	NA	7.0 (4.0, 8.5)		
Underlying				
Hypertension	0 (0.00)	9 (31.03)		
Diabetes	0 (0.00)	3 (10.34)		
Laboratory findings				
White blood cell, × 10^9^/L	5.77 (5.13, 6.17)	5.27 (4.21, 6.80)	0.96	0.335
Neutrophil, × 10^9^/L	3.28 (2.46, 4.02)	3.32 (2.49, 4.93)	0.50	0.619
Lymphocyte, × 10^9^/L	1.98 (1.61, 2.12)	1.07 (0.81, 1.49)	3.86	< 0.001
Hemoglobin, g/L	132 (122, 149)	144 (133, 151)	1.79	0.074
Platelet, × 10^9^/L	214 (182, 253)	176 (149, 198)	2.80	0.005
Fasting plasma glucose, mmol/L	NA	6.64 ± 2.23		
CD4 + T cell (μL)	NA	433 (272, 743)		
CD8 + T cell (μL)	NA	292 (171, 455)		

### Expression profiles of ISR-miRNAs at the acute phase of COVID-19

The relative expression levels of twelve ISR-miRNAs in the controls and COVID-19 patients during the acute phase were listed in Table 2. Compared with the healthy controls, COVID-19 patients exhibited higher expression levels of miR-29b-3p (Z = 3.15, *P* = 0.002) and miR-1246 (Z = 4.98, *P* < 0.001). However, the mean expression levels of miR-186-5p and miR-15a-5p in COVID-19 patients were significantly lower than those in controls. No significant differences in remaining ISR-miRNAs expression were observed between healthy controls and COVID-19 patients at the acute phase.

**Table 2:** Expression profiles of ISR-miRNAs in healthy controls and acute COVID-19 patients

ISR-miRNAs	Healthy controls (n = 29)	COVID-19 (n = 29)	Z	*P*
hsa-let-7c-5p	0.22 (0.05, 0.60)	0.21 (0.05, 0.44)	0.00	1.000
hsa-miR-29b-3p	0.06 (0.01, 0.25)	0.33 (0.11, 0.78)	3.15	0.002
hsa-miR-30b-5p	0.28 (0.10, 0.79)	0.58 (0.22, 1.60)	1.59	0.113
hsa-miR-186-5p	0.35 (0.17, 0.94)	0.06 (0.03, 0.19)	3.52	< 0.001
hsa-miR-15a-5p	0.13 (0.04, 0.53)	0.02 (0.01, 0.11)	2.26	0.024
hsa-miR-15b-5p	0.05 (0.02, 0.26)	0.06 (0.02, 0.13)	0.00	1.000
hsa-miR-148a-3p	0.12 (0.02, 0.29)	0.06 (0.03, 0.15)	0.99	0.322
hsa-miR-146b-3p	0.11 (0.03, 0.35)	0.15 (0.07, 0.27)	1.01	0.312
hsa-miR-409-3p	0.11 (0.03, 0.34)	0.24 (0.06, 0.34)	1.23	0.218
hsa-miR-497-5p	0.03 (0.01, 0.11)	0.05 (0.03, 0.12)	0.75	0.456
hsa-miR-548c-5p	0.06 (0.02, 0.28)	0.09 (0.04, 0.28)	1.44	0.151
hsa-miR-1246	0.06 (0.04, 0.11)	0.53 (0.19, 1.12)	4.98	< 0.001

### Association of ISR-miRNAs with clinical parameters at the acute phase of COVID-19

**Fig. 1:**
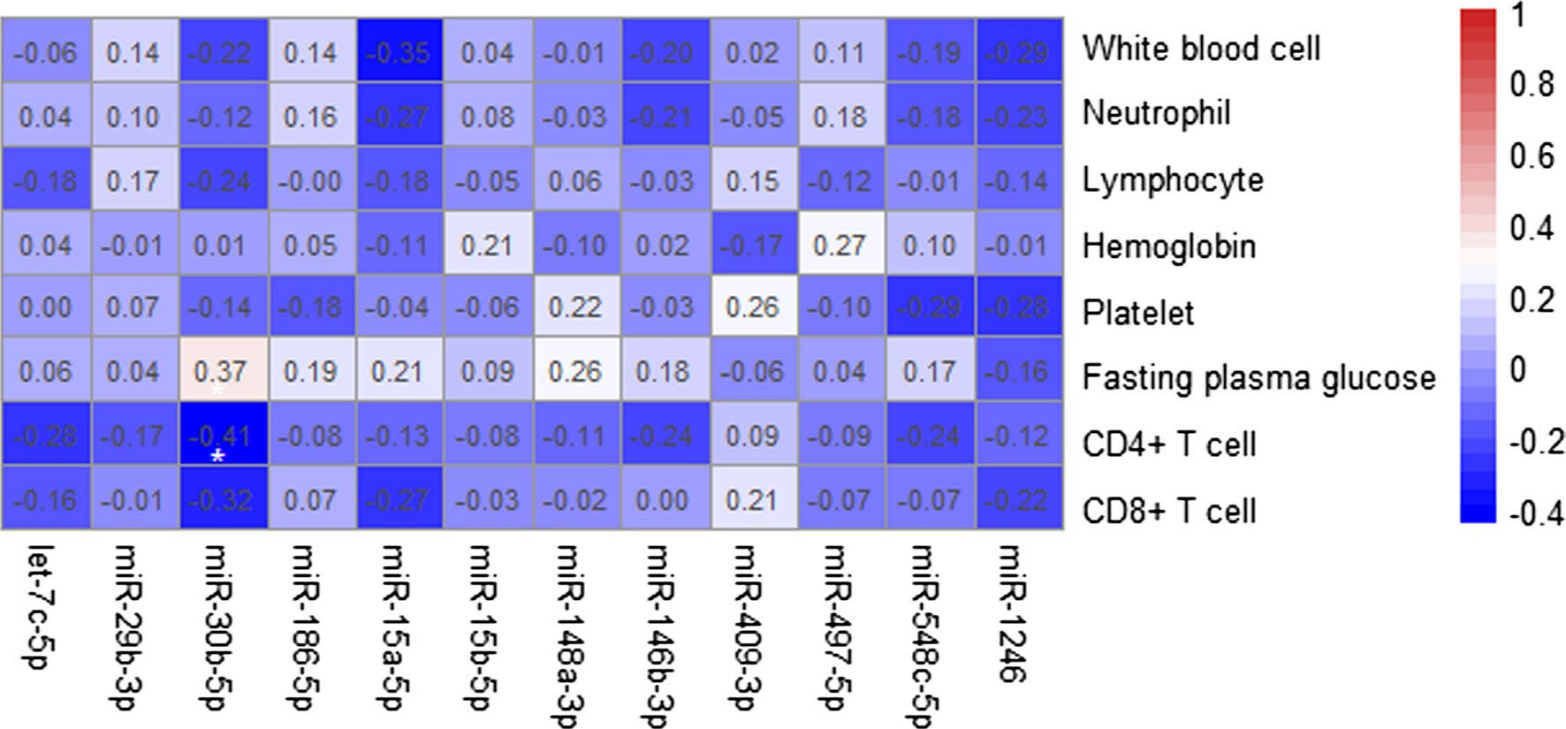
Spearman correlation between the relative expression levels of twelve ISR-miRNAs and clinical parameters. The values in each square are correlation coefficient (r) of each group samples. The dendrogram on the right reveals the sample’s correlation

The correlations between relative levels of ISR-miRNAs and clinically relevant parameters were exhibited in Fig. 1. The results showed that the plasma level of miR-30b-5p was negatively correlated with CD4 + T cell counts in patients with acute SRAS-CoV-2 infection (r = -0.41, *P* = 0.027). In addition, the relative concentration of miR-30b-5p was marginally correlated with fasting plasma glucose level (r = 0.37, *P* = 0.052). However, no significant correlation was observed between other ISR-miRNAs expression and clinically relevant parameters of COVID-19 patients at the acute phase.

### Association between ISR-miRNAs expression and RBD-IgG antibody response

Among 28 patients who provided enough convalescent plasma samples, RBD-IgG antibodies were detected in 27 COVID-19 patients using competitive ELISA. The highest and lowest PI values were 93.0% and 41.2% (median PI: 77.5%), respectively. As the results shown in Table 3, the counts of lymphocytes and CD8 + T cells at the acute phase of the disease was negatively correlated with the level of RBD-IgG antibody at the recovery phase, with r values of − 0.62 (*P* = 0.0004) and -0.43 (*P* = 0.023), respectively.

**Table 3:** Correlation between clinical parameters and RBD-IgG antibody response

	r	*P*
White blood cell	− 0.15	0.461
Neutrophil	0.12	0.541
Lymphocyte	− 0.62	0.0004
Hemoglobin	− 0.10	0.615
Platelet	− 0.18	0.368
Fasting plasma glucose	0.26	0.174
CD4 + T cell	− 0.29	0.138
CD8 + T cell	− 0.43	0.023

The association between the relative expression levels of plasma ISR-miRNAs at the acute phase of disease and the level of RBD-IgG antibody at the recovery phase was shown in Table 4. The plasma level of miR-497-5p at the acute phase of COVID-19 was positively correlated to RBD-IgG antibody response at the recovery phase of disease (r = 0.48, *P* = 0.038). However, no significant correlation was observed between other ISR-miRNA concentrations and RBD-IgG antibody response in the COVID-19 patients.

**Table 4:** Correlation between the expression levels of ISR-miRNAs and RBD-IgG antibody response

ISR-miRNAs	r	*P*
hsa-let-7c-5p	0.24	0.229
hsa-miR-29b-3p	− 0.08	0.689
hsa-miR-30b-5p	0.04	0.836
hsa-miR-186-5p	0.10	0.635
hsa-miR-15a-5p	0.28	0.257
hsa-miR-15b-5p	− 0.16	0.501
hsa-miR-148a-3p	− 0.06	0.778
hsa-miR-146b-3p	− 0.004	0.986
hsa-miR-409-3p	− 0.03	0.896
hsa-miR-497-5p	0.48	0.038
hsa-miR-548c-5p	0.09	0.676
hsa-miR-1246	0.04	0.850

## Discussion

Previously, several studies have indicated that ISR-miRNAs were significantly associated with SARS-CoV-2 infection [3–5]. For example, Centa A et al. reported that deregulated expression of miR-26a-5p, miR-29b-3p and miR-34a-5p was associated with endothelial dysfunction in post-mortem lung biopsies of COVID-19 patients [16]. Additionally, in a computational and bioinformatic analysis, Khan AT et al*.* speculated that miR-1246 might be upregulated in the respiratory distress caused by COVID-19 [17]. In the present study, our results further showed that the expression of miR-29b-3p and miR-1246 were significantly upregulated at the acute phase of COVID-19, suggesting that miR-29b-3p and miR-1246 might play an important role in the pathogenesis of acute SARS-CoV-2 infection.

Our present study observed that the expression levels of miR-186-5p and miR-15a-5p significantly decreased at the acute phase of COVID-19. Following in silico target prediction and pathway enrichment analyses, Zhao et al*.* suggested that miR-186-5p was depleted in retroviral infection. Similarly, Wu et al*.* reported that overexpressed miR-186 could inhibit the JAK/STAT signaling pathway in vitro [18]. In addition, the increased miR-186-5p expression could inhibit HIV infection by immunoregulation and T cell regulation [19]. The results from the most recent study also reported that miR-15b-5p were significantly downregulated in hamster lung samples infected by SARS-CoV-2 [20]. Considering that the miR-15 family members (i.e., miR-15a, 15b) possess the same seed sequence and have the same target genes, the present results further suggested that downregulated expression of miR-186-5p and miR-15a-5p might be helpful for the activation of IFN-I signaling pathway at the acute phase of SARS-CoV-2 infection.

Recently, Zheng et al*.* reported that the activation and differentiation of T cells are associated with various clinical features in patients with COVID-19 [21]. In a retrospective single-center study, Liu et al*.* further reported that CD4+ and CD8 + T cells were significantly related to the severity of SARS-CoV-2 infection [22]. Moreover, reduced CD3+, CD4+ and CD8 + T cell counts could reflect the severity of the COVID-19 [23]. In our study, the results showed the expression level of miR-30b-5p was negatively correlated with CD4 + T cell counts. These findings were shown from the side that miR-30b-5p might be associated with the severity of COVID-19 patients. Additionally, our present results showed that the level of miR-30b-5p increased with the level of fasting plasma glucose in COVID-19 patients at the acute phase. Considering that miR-30b-5p was much higher in patients with type 2 diabetes mellitus (T2DM) and the severity of COVID-19 was significantly associated with diabetes [24, 25], more studies should be conducted to clarify the effects of miR-30b-5p on SARS-CoV-2 infection in patients with and without T2DM.

Currently, a large number of studies have reported that the interaction between RBD located at the spike protein of SARS-CoV-2 and the receptor ACE2 on host cells is essential for viral entry [26, 27]. Moreover, antibodies against RBD at the recovery phase of COVID-19 present neutralizing activity because they can block the interaction between ACE2 and viral spike protein [28]. For example, Premkumar et al*.* reported that antibodies targeting RBD accounted for more than 90% of neutralizing activity in convalescent serum [29]. In this study, the results of competitive ELISA showed that 27/28 COVID-19 patients developed RBD-IgG antibodies at the recovery phase. Moreover, the plasma level of miR-497-5p at the acute phase positively correlated with RBD-IgG antibody response. However, the counts of lymphocyte and CD8 + T cells during the acute phase were inversely correlated with RBD-IgG antibody response, indicating that the mechanisms of miR-497-5p expression involved in RBD-IgG response should be further explored.

Our present study has several limitations. First, considering that only 29 patients were included in this study, caution should be taken when interpreting the present findings. Second, since the disease progression and antibody response might be influenced by various risk factors (age, comorbidity disease such as hypertension and diabetes) [30–32], the effects of the ISR-miRNAs and interactions of other risk factors on SARS-CoV-2 infection should also be carefully verified in future. Last, although our study was the first time to report that miR-29b-3p, miR-1246, miR-186-5p and miR-15a-5p expressions were significantly altered during acute SARS-CoV-2 infection, the difference in plasma ISR-miRNAs between patients with mild and severe infection could not be clarified because of the limited sample size.

## Conclusions

The expression level of ISR-miRNAs was not only associated with acute SARS-CoV-2 infection but also with RBD-IgG antibody response at the recovery phase of the disease. However, considering that the limited sample size in this study, further studies including more patients are needed to validate our present findings and to explore the biological significance of ISR-miRNAs during SARS-CoV-2 infection.

## Supplementary Information

**Additional file 1: Table S1.** List of human miRNAs with target gene and miRNAs seed binding site on COVID-19 isolates.

**Additional file 2: Table S2.** Product number of the primers for ISR-miRNAs quantitation. Footnote: RiboBio Corporation (Guangzhou, China), https://www.ribobio.com/.

## Data Availability

The data and materials included in the present study could be provided by Chen Dong (cdong@suda.edu.cn) and Jing Wu (20194247006@stu.suda.edu.cn) on reasonable request.

## Abbreviations

COVID-19: Coronavirus disease 2019
SARS-CoV-2: Severe acute respiratory syndrome coronavirus-2
IFN-I: Type I interferon
JAK-STAT: Januskinase/signal transducer and activator of transcription
ISGs: IFN-stimulated genes
HCV: Hepatitis C virus
microRNA: MiRNA
ISR-miRNAs: IFN-I signaling-related miRNAs
CT: Cycle threshold
OD: Optical density
RBD: Receptor-binding domain
PI: Percent inhibition
T2DM: Type 2 diabetes mellitus
