# Supplementary material for: Expression of plasma IFN signaling-related miRNAs during acute SARS-CoV-2 infection and its association with RBD-IgG antibody response
Source: Virol J. 2021 Dec 7;18:244. doi: 10.1186/s12985-021-01717-7 (PMC8649682; doi:10.1186/s12985-021-01717-7)
Supplement: Supplementary file 2 — Additional file 2: Table S2. Product number of the primers for ISR-miRNAs quantitation. Footnote: RiboBio Corporation (Guangzhou, China), https://www.ribobio.com/. [file 12985_2021_1717_MOESM2_ESM.docx]

Table S2 Product number of the primers for ISR-miRNAs quantitation

| ISR-miRNAs | Product number of primer^#^ |
| --- | --- |
| hsa-let-7c-5p | miRA0000064 |
| hsa-miR-29b-3p | miRA0000100 |
| hsa-miR-30b-5p | miRA0000420 |
| hsa-miR-186-5p | miRA0000456 |
| hsa-miR-15a-5p | miRA0000068 |
| hsa-miR-15b-5p | miRA0000417 |
| hsa-miR-148a-3p | miRA0000243 |
| hsa-miR-146b-3p | miRA1000403 |
| hsa-miR-409-3p | miRA1000089 |
| hsa-miR-497-5p | miRA1000091 |
| hsa-miR-548c-5p | miRA1000420 |
| hsa-miR-1246 | miRA1000125 |

# RiboBio Corporation (Guangzhou, China), https://www.ribobio.com/
